# Symptom prevalence of patients with fibrotic interstitial lung disease: a systematic literature review

**DOI:** 10.1186/s12890-018-0651-3

**Published:** 2018-05-22

**Authors:** Sabrina Carvajalino, Carla Reigada, Miriam J. Johnson, Mendwas Dzingina, Sabrina Bajwah

**Affiliations:** 1 Fundación Santa Fé de Bogotá, Bogotá, Colombia; 2 Hull York Medical School, Hertford Building, University of Hull, Hull, UK; 3 Cicely Saunders Institute, Bessemer Rd, London, UK

**Keywords:** Pulmonary fibrosis, Symptom prevalence and interstitial lung disease

## Abstract

**Background:**

Those affected by advanced fibrotic interstitial lung diseases have limited treatment options and in the terminal stages, the focus of care is on symptom management. However, quantitatively, little is known about symptom prevalence. We aimed to determine the prevalence of symptoms in Progressive Idiopathic Fibrotic Interstitial Lung Disease (PIF-ILD).

**Methods:**

Searches on eight electronic databases including MEDLINE for clinical studies between 1966 and 2015 where the target population was adults with PIF-ILD and for whom the prevalence of symptoms had been calculated.

**Results:**

A total of 4086 titles were screened for eligibility criteria; 23 studies were included for analysis. The highest prevalence was that for breathlessness (54–98%) and cough (59–100%) followed by heartburn (25–65%) and depression (10–49%). The heterogeneity of studies limited their comparability, but many of the symptoms present in patients with other end-stage disease were also seen in PIF-ILD.

**Conclusions:**

This is the first quantitative review of symptoms in people with Progressive Idiopathic Fibrotic Interstitial Lung Diseases. Symptoms are common, often multiple and have a comparable prevalence to those experienced in other advanced diseases. Quantification of these data provides valuable information to inform the allocation of resources.

**Electronic supplementary material:**

The online version of this article (10.1186/s12890-018-0651-3) contains supplementary material, which is available to authorized users.

## Background

Patients with Interstitial Lung Disease have a wide range of diagnoses and prognoses. Many patients can live many years with their diagnosis and some are responsive to treatments. However, a subset of patients with Progressive Idiopathic Fibrotic Interstitial Lung Diseases (PIF-ILD) such as idiopathic pulmonary fibrosis have a short disease trajectory and a similar prognosis to people with lung cancer [1]. The clinical manifestation of advanced fibrotic Non Specific Interstitial Pneumonia (NSIP) is similar to IPF [2]. It is important to differentiate NSIP from IPF in the early stages when the disease is potentially responsive to therapy [2] .However, when the disease is advanced and irreversible, this becomes less important and the focus should be on symptom control.

The United Kingdom (UK) End of life care strategy aimed to promote high quality care for all adults at the end of life [3]. In addition, the British Thoracic [4] and NICE idiopathic pulmonary fibrosis guidance [5] emphasize the importance of a proactive approach in managing symptoms.

Recent qualitative work in this group has shown uncontrolled symptoms, for example, shortness of breath, cough and insomnia, which impact on every aspect of patients and carers lives [6, 7]. However, quantitative work assessing prevalence of symptoms is limited and there has been no systematic review of this literature. Synthesising the quantitative evidence for symptom prevalence for this group will add to previous qualitative work, raise awareness of these symptoms and focus clinical intervention.

## Methods

### Aim

To estimate the symptom prevalence in people with PIF-ILD.

### Design

Systematic review of the literature.

### Search strategy

We performed comprehensive searches of databases including MEDLINE, Cochrane, EMBASE, Science Citation Index Expanded (Web of Knowledge), pre-Medline, CINAHL and PSYCINFO from 1966 to November 2013 using a combination of MESH headings and keywords (for full search strategy see online Additional file 1 APPENDIX A). In addition, key journals hand searched included THORAX, American Journal of Respiratory and Critical Care Medicine and CHEST (2000 to 2013). The search was updated to March 2015. Only studies in English or Spanish were included.

### Selection

#### Study population

Published data of adults (≥ 18 years old), with all stages of the following ILD types: interstitial pulmonary fibrosis (IPF), nonspecific interstitial pneumonia (NSIP), cryptogenic fibrosing alveolitis and idiopathic interstitial pneumonia from any setting were included.

Studies in which patients had COPD and/or cancer in addition to PIF-ILD were excluded.

#### Types of studies included

A scoping search identified a paucity of data. Therefore all study types reporting quantitative data were included. Case reports of fewer than five patients were excluded. Qualitative studies were included if quantitative data were available for extraction.

#### Types of outcomes included

Symptoms included were based on a previous systematic review looking at interventions to improve symptoms and quality in patients with PIF-ILD [8] and encompassed both physical and psychological domains.

### Data extraction

One independent reviewer (SC) selected the studies against the inclusion criteria using the title and, if the title did not offer enough information, abstracts and/or full text were read. Data were extracted using a form that included the main author, year of publication, setting, type and number of participants, disease group, aims of the study, study design, measurement methods and prevalence of individual symptoms (See Additional file 2 APPENDIX B).

### Data analysis

The Strengthening the Reporting of Observational Studies in Epidemiology (STROBE) Statement checklist for observational studies [9] was used to appraise each of the final studies. A palliative symptom grid was used and the number of patients in each study was calculated for each of the symptoms. Meta-synthesis and descriptive statistics were used for analysis and to present the findings. Where appropriate, a meta-analysis of each symptom from multiple studies was conducted using a random-effects model with inverse-variance weighting. Symptoms which were reported in only two studies or less were excluded from the meta-analysis. Heterogeneity was also quantified using the I-squared measure [10]. The confidence intervals are based on exact binomial (Clopper-Pearson) procedures [11]. Meta-analysis was conducted in Stata (StataCorp 2015) release 14 [12].

## Results

### Overview of included studies

**Fig. 1:**
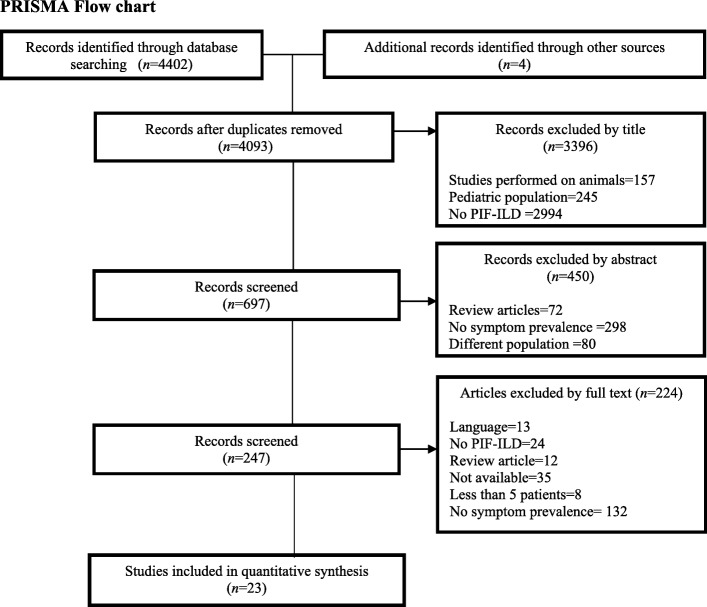
Pooled estimates of prevalence (proportion) of symptoms- random effects model ES = Estimated proportion

Twenty-three articles describing symptoms were selected for this review (see Fig. 1) potentially relevant but excluded studies have been listed separately in Additional file 3 APPENDIX C. Included studies represented *N* = 3171 patients from European, Asian, and North and South American countries, conducted on outpatients at different disease stages; four studies included patients with end-stage disease [13–16]. The mean age across all studies varied between the fifth and sixth decade of life, and one study included patients older than 65 years [17]. Overall, studies found prognosis ranged between 12.9 to 46 months from time of diagnosis.

Study designs varied with a variety of retro and prospective designs (Table 1).

**Table 1:** Summary of studies included

Author/Year	Aim	Study Design and symptom assessment used	Participants (n)	Diagnosis	Diagnosis method	Baseline % predicted lung function mean (SD)	Symptoms prevalence
Akhtar 2013 [33]	To assess the presence of depressive symptoms	Prospective study Wakefield Self-assessment of Depression Inventory score ≥ 15 screening tool	Outpatients (*n* = 118)	IPF	High resolution computed tomography, Lung biopsy	Not available	Depression 49.2%
Alhamad 2008 [18]	Describe the clinical course and prognosis of IPF among Middle Eastern patients, and to attempt to identify variables that would predict prognosis.	Retrospective study Chart reviews, telephone interviews	Hospital patients (*n* = 61)	IPF	ATS/ERS criteria	FVC 64.8 (21.6)^a^	Dyspnoea 93%; Cough 82%; Weight loss 12%
Araki 2003 [17]	To investigate the outcome of IPF in elderly patients whose pathological diagnosis corresponded to usual interstitial pneumonia on autopsy findings.	Retrospective study MRC dyspnoea scale, medical records	Patients older than 65y, based on histological findings on autopsy, complete medical records (*n* = 86)	UIP, IPF	Lung biopsy: Histological findings consistent with UIP, IPF	VC 72.6 (25.2) DLCO 62.8 (30.1)	Dyspnoea 54.7% Cough 93.2%
Bajwah 2012 [19]	To compare the palliative care needs, treatments, and end-of-life preferences of PIF-ILD patients	Retrospective study Medical records	Outpatients Hospital; Ages 37–99 (*n* = 45)	PIF-ILD	ATS/ERS criteria	Not available	Dyspnoea 93%; Cough 60%; Fatigue 29%; Insomnia 6%; Depress ion/anxiety 22%; Anorexia/weight loss 18%; Chest pain 29%; Generalized pain 9%; Dyspepsia 4%; Polyuria/polydipsia 4%; Diarrhea 2%; Dysphagia 2%
Bandeira 2009 [20]	To determine prevalence of GERD and to evaluate its clinical presentation	Prospective study General questionnaire, Quality of Life Scale for Gastroesophageal Reflux Disease	Outpatients (*n* = 28)	IPF	ATS/ERS criteria in 11 patients, lung biopsy 17 patients	FVC 66.6 (16.0) DLCO 44.5 (22.0)	Heartburn 29%; Nocturnal heartburn 14%; Regurgitation 40%; Nocturnal regurgitation 18%; Epigastric pain 18%; Dysphagia 11%; Cough 77%; Nocturnal cough 37%; Dysphonia 11%; Chest pain 25%
D’Ovidio 2005 [13]	To determine the prevalence of gastroesophageal reflux in lung transplant candidates	Interviews and Esophageal manometer.	Outpatients (*n* = 26)	IPF	Not specified	FVC median (range) 67 (33–96) DLCO median (range) 40 (13–77)	Heartburn Regurgitation Dysphagia 65%
Hashemi Sadraei 2013 [21]	To evaluate the clinical characteristics of IPF patients from The National Research Institute of Tuberculosis and Lung Diseases	Retrospective descriptive study Medical records and interviews	(*n* = 132)	IPF	Clinical presentation, radiographic and or/pathological findings ATS criteria	Not available	Breathlessness 68.2%; Cough 60.6%; Chest pain 8.3%; Fatigue 7.6%
Hoppo 2012 [14]	To determine the prevalence of GERD and assess the proximity of reflux events in patients with histologically proven IPF	Retrospective study	(*n* = 35)	IPF	Lung biopsy	Not available	Cough 74%; Heartburn 25%; Regurgitation 25%
Jeon 2006 [22]	To investigate the prognostic factors at initial presentation and the causes of death in Korean patients with IPF	Retrospective study Medical records	Outpatients (*n* = 88)	IPF	Surgical lung biopsy compatible with UIP, ATS criteria	FVC 74.0 (19.2) DLCO 65.2 (21.4)	Exertional dyspnoea 89%
Lancaster 2009 [31]	To analyze obstructive sleep apnea in clinically stable patients with IPF	Epworth sleepiness scale (ESS) ≥10 consistent with daytime sleepiness	(*n* = 35)	IPF	ATS criteria (2000)	FVC 68.8 (13.7)^a^	Daytime sleepiness 25%
Lindell 2010 [34]	To test the ability of a complex intervention (PRISM) to decrease symptom burden, stress and improve HRQoL perceptions of patients with IPF and their carers.	Nested mixed method design (experimental, qualitative) Beck Anxiety Inventory, Beck Depression Inventory-II	Outpatients (*n* = 37)	IPF	Biopsy and/or High resolution computed tomography	70% FVC > 55 15% FVC 50–55 15% FVC < 50%	Anxiety 58% Depression 4 (10%)
Mermigkis 2009 [32]	To describe sleep quality associated to daytime consequences in IPF	Cross-sectional control study Epworth Sleepiness Scale Pittsburgh Sleep Quality Index Functional Outcomes in Sleep Questionnaire Fatigue Severity Scale Polysomnography Interview	Outpatients (*n* = 15)	IPF	ATS/ERS criteria or lung biopsy	FVC 77.4 (21.2) DLCO 56.3 (17.8)	Daytime sleepiness 20%; Snoring 40%; Insomnia 46.6%; Witnessed apnoea’s 13.3%
Mermigkis 2007 [28]	To describe the clinical and polysomnographic features of SRBD and to identify predictors of OSA in IPF patients	Retrospective study Cleveland Clinic Sleep Disorders Questionnaire, Epworth Sleepiness scale, Polysomnography	Outpatients (*n* = 18)	IPF	ATS/ERS criteria	FVC 65.7 (10.4) DLCO 49.9 (15.3)	Excessive daytime sleepiness 77.7%; Snoring 88%; Daytime fatigue 61%; Witnessed apnoea’s 44.4%
Ohno 2007 [23]	Not specified	Retrospective Clinical personal records	(*n* = 1322) Patients covered by public insurance	IIP	Medical records: 12% pathological diagnosis from lung biopsy, rest clinical findings (respiratory function test, images, serology)	Not available	Cough 94%; Exertional dyspnoea 98%
Patti 2005 [15]	To determine the prevalence of GERD, the clinical presentation of GERD and reflux profiles in patients with IPF	Patients rated severity of symptoms 5 point scale (0 = no symptom to 4 = disabling symptom)	Outpatients (*n* = 18)	IPF	Not specified	Not available	Heartburn 55%; Regurgitation 33%; Cough 83%
Raghu 2006 [29]	To assess the prevalence and clinical symptoms of GER in patients with IPF and compare findings to patients with intractable asthma manifesting symptoms of GER.	Prospective study 24 h oesophageal pH probe, oesophageal manometry, symptom questionnaire form	Outpatients (*n* = 65)	IPF	ATS criteria	FVC 59.9 (20.0)^a^ DLCO 34.8 (15.7)^a^	Belching 51%; Heartburn 47%; Regurgitation 16%; Abdominal pain 7%; Bloating 27%; Chest pain 24% Choking 13%; Globus 13%; Hoarseness 31%; Liquid dysphagia 7%; Solid dysphagia 16%; Odynophagia 4%; Nausea 13%
Ryerson 2012 [35]	To investigate the prevalence of clinically meaningful depress ion at baseline, characterize the association of depression with patient and disease specific variables, and describe the natural history of depress ion over a period of 6 months	Cohort	Outpatients (n=)52	ILD (21 with IPF)	ATS/ERS criteria	FVC 74.3 (18.5) DLCO 50.8 (16.3)	Depression 24%
Schoenheit 2011 [24]	To generate in depth insights regarding the patient journey, including symptoms triggers to seeking medical care, referral patterns, initial diagnoses, follow up and current disease management.	Qualitative Interviews conducted in the participants at home	Outpatients (*n* = 45)	IPF	Physician confirmed diagnosis	Not available	Exertional dyspnoea 68%; Cough 59%; Fatigue 28%; Chest pain 6%; Weight loss 2%
Sweet 2007 [16]	To determine the prevalence of distal and proximal reflux, the oesophageal manometric profile and whether or not reflux symptoms could be used to screen for reflux	Retrospective Study Standardized interview with a physician or technician. Patients rated severity of symptoms 5 point scale (0 = no symptom to 4 = disabling symptom)	Outpatients (*n* = 30)	IPF	Pathological findings in 25 patients, ATS/ERS criteria in 5 patients	Not available	Heartburn 48%; Regurgitation 43%; Dysphagia 30%
Tobin 1998 [25]	To investigate the possible association of GER and IPF	Qualitative study Structured interview	Outpatients (*n* = 17)	UIP	Lung biopsy compatible with UIP	DLCO mean (range) 35.9 (9–62)	Cough 100%
Von Plessen 2003 [26]	To study the incidence and prevalence of physician diagnosed and hospitalized cryptogenic fibrosing alveolitis in a well-defined adult population in Norway	Retrospective study Registration form, hospital registers (2 physicians extracted the information)	Hospital patients 158 incident cases (1984–1998) and 61 prevalent cases (until 31.12. 1998)	CFA	Progressive dyspnoea, crackles on auscultation and bilateral shadowings on chest X-ray with no exposure to a known fibrogenic agent	83 and 80% of incident and prevalent cases TLCO < 80% predicted	Incident cases dyspnoea 87%; Prevalent cases 79%
Aksu 2014 [30]	To investigate the possibility that IPF is involved in the pathogenic of GERD	Prospective study	Outpatients (*N* = 21)	IPF	Pulmonary function tests (spirometry, carbon monoxide diffusion capacity, alveolar volume), study of BAL fluid (cell count and lymphocyte subsets, IL-1 β, TNF-α)	FVC 94.9 (11.2)^a^ TLCO 114.1 (16.7)	Reflux symptoms 52.4% Severe dysphagia 23.8% Epigastric pain 91%
Huang 2014 [27]	To describe the clinical features and prognosis of microscopic polyangiitis (MPA) patients whose initial respiratory presentation was pulmonary fibrosis	Retrospective study Hospital computer-assisted search	Hospital patients MPA cases (*N* = 67)	IPF patients (*N* = 19)	Radiological findings (CT), clinical manifestations consistent with UIP pattern according to the ATS/ERS/JRS/ALAT statement 2011	DLCO range 30–76	Of IPF patients: Cough 84.2% Sputum 68.4% Hemoptysis 21.1% Dyspnoea 78.9%

^a^ mean estimates were pooled using the inverse variance weighting method

### Symptom prevalence

**Fig. 2:**
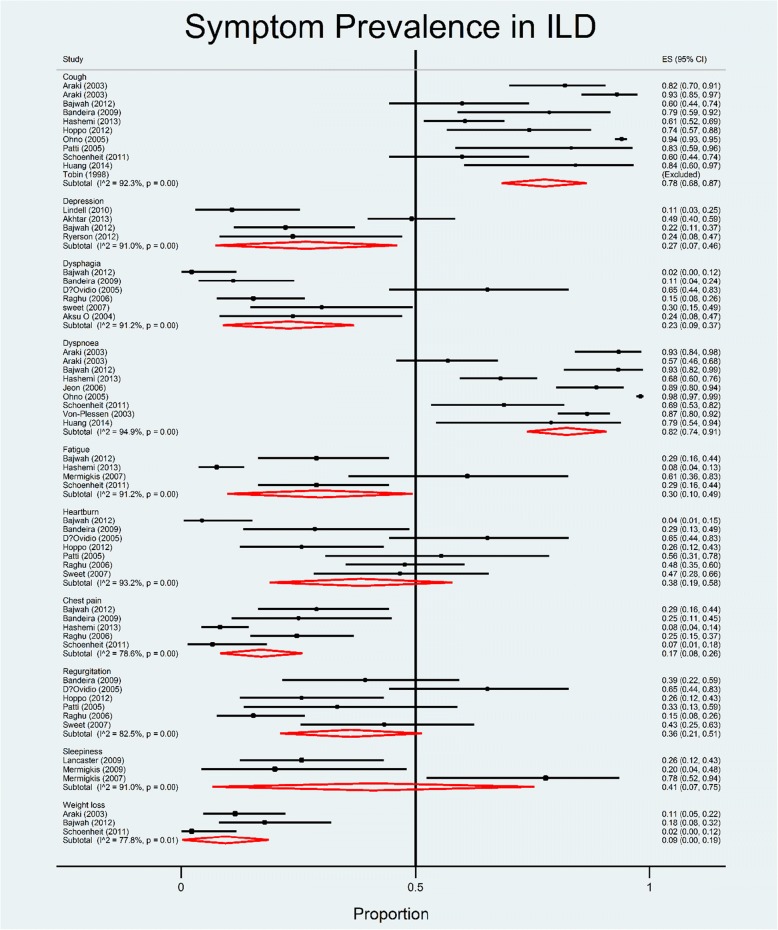
Pooled estimates of prevalence (proportion) of symptoms- random effects model. ES = Estimated proportion

Respiratory symptoms such as breathlessness and cough were measured in 13 studies [14, 15, 17–27]; fatigue and weight loss in five [18, 19, 21, 24, 28]; digestive tract symptoms in eight [13–16, 19, 20, 29, 30] sleep disorder in four [19, 28, 31, 32]; and other symptoms such as pain and urinary tract disorders in five [19–21, 24, 29]. The incidence of depression and/or anxiety was calculated in four studies [19, 33–35]. No studies documented delirium, constipation, halitosis, hemoptysis, hiccups, hyperphagia, polydipsia or mouth problems. A summary of findings is presented in Fig. 2.

#### Respiratory symptoms

The overwhelming majority of patients had breathlessness (68.2–98%) and cough (59–94%) [14, 15, 17–27]. These were not only common symptoms, but preceded diagnosis by 6.8 months to 4 years [18]. Only one study documented breathlessness using the modified Medical Research Council scale (mMRC) (range 0 to 4) in 45.3% of the participants [17]. Nearly one in ten (9.3%) had mMRC grade 4. Severe breathlessness was associated with poor prognosis and those with mMRC scale score 3 and 4 had a median survival of 0.5 years [17].

#### Depression

A variety of depression measurement tools were used to provide prevalence estimates of depression ranking between 10% [34] and 49.2% [19, 33, 35]. A worse depression score was found to be associated with reduced Forced Expiratory Volume (FEV), Forced Vital Capacity (FVC), gas transfer factor and gas constant, increased duration of diagnosis, greater number of comorbidities [33]; worse breathlessness severity, pain severity, sleep quality, 4-m walk time, grip strength and diffusing capacity of the lung for carbon monoxide (DLco) [35].

The prevalence of anxiety was estimated to be as high as 58% in one study of health-related quality of life (HRQoL) and symptom burden [34].

#### Digestive tract symptoms

Upper gastro-intestinal symptoms are described in IPF and have been investigated in several studies. Gastro-oesophageal reflux prevalence was shown in 35.7 to 100% [13–16, 20, 30].

Although in some patients this appeared to be asymptomatic, symptoms were reported by a significant proportion: belching (51%) [29], regurgitation (16–40%) [13–16, 20, 29], heartburn (29–48%) [13–16, 20, 29], dysphagia (11–43%) [13, 16, 20, 29, 30], and dysphonia (11%) [20]. Typical acid reflux symptoms were found [13–15, 20] and usually related to other causes such as cough (83% in these studies). A correlation between cough and acid reflux in the oesophagus was seen in 28% of the episodes of reflux [25]. However, 33% of those without evidence of dysmotility had at least one oesophageal symptom [20].

#### Sleep related symptoms

A relationship between obstructive sleep apnoea and IPF was observed in a 50 patients study with stable breathlessness, which a quarter of participants had an Epworth sleepiness score higher than 10 representing significant daytime sleepiness [31]. In one study of 30 patients, the following sleep related symptoms were reported: insomnia (46.6%), snoring (40%), excessive daytime sleepiness (20%), and witnessed apnoea’s (13.3%) [32]. Studies used variety of outcome measures and showed problems with daytime fatigue (The Functional Systems Scores (FSS)), daytime dysfunction (Functional Outcomes of Sleep Questionnaire (FOSQ)) and poor sleep quality (Pittsburgh Sleep Quality Index (PSQI)). Patients reported excessive daytime sleepiness (77.7%), snoring (88%), daytime fatigue (29%), witnessed apnoeas (44.4%), and insomnia (6–46%) [19, 28, 31, 32].

#### Anorexia, weight loss, fatigue

The prevalence of weight loss was estimated as 2–18% (out of *N* = 151), and the prevalence of fatigue as 7.6–29% out of *N* = 240 [18, 19, 21, 24, 28].

#### Pain

Non-specified pain was found in 9% of the population, while chest pain affected 6–29% [19–21, 24, 29]. Two studies found epigastric pain in 18 and 91% of the population [20, 30].

#### Other symptoms

A prevalence of polyuria/polydipsia prevalence of 4% was found in one study [19].

## Discussion

This is the first systematic review to draw together the symptom profile of people with PIF-ILD and shows a wide array of symptoms; comparable with those reported in other advanced diseases [36] (see Table 2). Breathlessness is seen to be a major problem, as prevalent as for people with COPD and heart disease. Likewise, psychological problems (depression and anxiety) and insomnia are prevalent in PIF-ILD. However, given the comparable high prevalence of both breathlessness, anxiety and sleep disturbance, the estimate for daytime fatigue was surprisingly low [28]. This may be explained, at least in part, by the different outcome measures used to assess sleep quality in the different studies and only one study accounted for comorbid conditions that might interfere with sleep quality and quality of life [28].

**Table 2:** Summary of the prevalence of symptoms in Cancer, AIDS, CHF, COPD, ESRD and PIF-ILD (figures for other conditions taken from Solano et al. 2006 [36])

Symptoms	PIF-ILD	Cancer	AIDS	CHF	COPD	ESRD
Pain	9%	30–94%	30–98%	14–78%	21–77%	11–93%
Depression	10–49.2%	4–80%	17–82%	6–59%	17–77%	2–61%
Anxiety	22–58%	3–74%	13–76%	2–49%	23–53%	7–52%
Fatigue	7.6–29%	23–100%	43–95%	42–82%	32–96%	13–100%
Breathlessness	54.7–98%	16–77%	43–62%	18–88%	56–98%	11–82%
Insomnia	6–46.6%	3–67%	40–74%	36–48%	15–77%	1–83%
Nausea	13%	2–78%	41–57%	2–48%	4%	8–52%
Diarrhea	2%	1–95%	29–53%	12%		8–36%

*AIDS* Adult Immune Deficiency Syndrome, *CHF* Chronic Heart Failure, *COPD* Chronic Obstructive Pulmonary Disease, *ESRD* End-stage Renal Disease

Two other symptoms stand out as particular problems for people with PIF-ILD. Firstly, cough is identified as not only highly prevalent, but also of major significance in terms of symptom burden, often preceding the diagnosis by some time. Secondly, although reports of nausea and vomiting are relatively low, there are significant problems associated with gastro-intestinal dysmotility leading to reflux which is likely to aggravate cough and may be associated with chest/epigastric pain.

Most people with respiratory disease have multiple co-morbidities which contribute long- term symptoms [37]. In addition, symptoms do not occur in isolation with demonstrated interactions between many symptoms, particularly in lung cancer, where a respiratory distress cluster of cough, breathlessness and fatigue has been described [38, 39]. The possibility of specific symptom clusters (clinically observed symptoms associations) for PIF-ILD which could benefit from a combined symptomatic approach is an area for further research. Knowledge of symptom clusters in PIF-ILD may help prompt clinical investigation of associated symptoms when one symptom is detected. It is clear from these data that a single symptom does not occur in isolation. Therefore is important that symptom assessment in people with PIF-ILD should focus on all commonly encountered symptoms and not just breathlessness alone. The significant prevalence of anxiety, depression and social isolation as the disease progresses highlights the importance of a holistic approach embodied by palliative care [6].

Palliative care is the active, total care of people with advanced, progressive disease [40]. Currently, the vast majority of palliative care services are provided to patients with cancer, and access to specialist palliative care is inconsistent for people with non-malignant disease. This inequity has been highlighted in the recent NICE guidance for IPF [5]. These stated that the ILD specialist services should have the skills to assess and manage most supportive and palliative care needs of the people under their care. In addition, robust joint working and pathways of care should also be in place to ensure access to specialist palliative care for those issues that the ILD services are unable to address. However, this policy has been largely unimplemented, and in everyday practice as currently configured, patients have unmet palliative care needs [7, 41].

### Implications for clinical practice and research

People with PIF-ILD face a sombre prognosis and deterioration in their quality of life with little hope of successful disease modification. Therefore, improvement in quality of life and palliation of significant symptoms are crucially important treatment goals [5, 42]. Recognition that these are prevalent is the first step, the next is to incorporate systematic assessment of symptoms and other palliative care concerns as a routine part of clinical management by respiratory health professionals. There needs to be a recognition that other symptoms alongside breathlessness are present. This is likely to have implications for education and training needs, extended team working between respiratory, palliative and primary care, and service configuration. Validated clinical tools to aid the clinician to identify and triage symptoms and other needs are needed for everyday practice and has been highlighted in the recent NICE quality standard for IPF [42]. An example of such tool is the recently adapted and validated Needs Assessment Tool-Interstitial Lung Disease (NAT-ILD) [43].

Good quality prospective observational studies are needed to get better estimates of symptom prevalence in PIF-ILD over the duration of the disease. Such prospective evaluation would allow investigation of symptoms not found in this review such as confusion, constipation and anorexia. In particular, the natural history of symptoms as the disease progresses to advanced disease and end of life along with the impact upon the individual and their family needs to be described in order to be able to understand the clinical care needs of this patient group, inform palliative and supportive care service planning and to inform study designs for clinical trials of symptom interventions. To facilitate this, disease severity with baseline lung function should be published for all studies.

Symptoms which seem to be of particular concern to people with PIF-ILD such as cough and gastro-intestinal dysmotility are under-researched and deserve focus. In addition, validation of questionnaires to determine the presence of conditions such as depression and fatigue in this group would be useful.

### Limitations

Only one reviewer screened, selected and extracted data from the articles included. Those not published in the English or Spanish language were excluded. Grey literature was not searched. It was difficult to give an accurate estimate of symptom prevalence due to the varying quality of cohort formation, measurement tools and definition of the symptom in question. Period prevalence time ranges, varying definitions of symptoms, sample size proportions and the various different measurement methods across the studies may all have contributed to variations in the minimum and maximum prevalence ranges. Due to the heterogeneity of the study populations and poor reporting, meta-analyses and sub-analyses by disease and severity of disease was not possible. Patients included in these studies had stable disease and were not receiving oxygen therapy.

## Conclusion

This study aimed to determine from existing studies, the prevalence of a group of symptoms in patients with PIF-ILD. Symptoms are common, often multiple and have a comparable prevalence to those experienced by people with other advanced diseases. Symptoms extend far beyond respiratory symptoms such as breathlessness and cough, and include fatigue, sleep disturbance as well as a broad variety of gastrointestinal symptoms. Breathlessness and anxiety are as prevalent as in COPD and heart disease, yet patients rarely have access to breathlessness management programs. Cough and gastrointestinal dysmotility appear to be particular issues for people with PIF-ILD and warrant further work which should include exploration of a possible PIF-ILD symptom clusters.

Quantification of these symptoms provide valuable information to inform the education and training needs of ILD services to allow routine assessment and management by ILD clinicians and appropriate use and allocation of specialist palliative care resources.

These findings highlight and support the need for a systematic and validated approach to assessment of symptoms in every day clinical practice by ILD services. This would ensure close attention to symptom management with appropriate and timely referral to palliative care services according to need, in order to optimise quality of life and provide good care during advanced disease and end of life.

## Additional files

Additional file 1: Appendix A Full search strategy – Medicine search strategy. (DOCX 14 kb)

Additional file 2: Appendix B Data extraction form. (DOCX 13 kb)

Additional file 3: Appendix C Potentially Relevant but Excluded Studies. (DOCX 12 kb)

## Abbreviations

DLco: Diffusing capacity of the lung for carbon monoxide
FEV: Forced Expiratory Volume
FOSQ: Functional Outcomes of Sleep Questionnaire
FSS: Functional Systems Scores
FVC: Forced Vital Capacity
HRQoL: Health-related quality of life
mMRC: Modified Medical Research Council scale
MPA: Microscopic polyangiitis
NAT-ILD: Needs Assessment Tool-Interstitial Lung Disease
NSIP: Non Specific Interstitial Pneumonia
PIF-ILD: Progressive Idiopathic Fibrotic Interstitial Lung Disease
PSQI: Pittsburgh Sleep Quality Index
STROBE: Strengthening the Reporting of Observational Studies in Epidemiology
