# Supplementary material for: Symptom prevalence of patients with fibrotic interstitial lung disease: a systematic literature review
Source: BMC Pulm Med. 2018 May 22;18:78. doi: 10.1186/s12890-018-0651-3 (PMC5964639; doi:10.1186/s12890-018-0651-3)
Supplement: Supplementary file 1 — Appendix A Full search strategy – Medicine search strategy. (DOCX 14 kb) [file 12890_2018_651_MOESM1_ESM.docx]

Additional file 1 Appendix A Medline search strategy

1. exp idiopathic interstitial pneumonias

2. Fibrotic interstitial lung disease

3. Idiopathic pulmonary fibrosis

4. Interstitial pulmonary fibrosis

5. Fibrotic non-specific interstitial pneumonia

6. Fibrotic NSIP

7. Fibrotic lung disease

8. Pulmonary fibrosis

9. IPF

10. Cryptogenic fibrosing alveolitis

11. Interstitial pneumonia

12. UIP

13. IIP

14. Terms 1-13 were combined using OR

15. Anorexia or loss adj2 appetite

16. Anxiety or anxiety disorders or anxious

17. Breath*

18. Confusion

19. Constipation

20. Cough

21. Delirium

22. Depression or depressive disorders or mood disorders or depressive symptoms or low adj mood

23. Diarrhea or diarr$

24. Dyspepsia or acid reflux

25. Dysphonia

26. Dyspnea or dyspnoea or dyspnoeic

27. Emesis or vomiting

28. Fatigue or tiredness or weakness

29. Flatulence

30. Gagging

31. Halitosis

32. Heart burn

33. Hemoptysis or h$moptysis

34. Hiccup or hiccough

35. Hyperphagia

36. Hoarseness

37. Nausea

38. Organic brain syndrome

39. Pain

40. Polyuria

41. Polydipsia or thirst

42. Sleep initiation and maintenance disorders or sleeplessness or problem$ sleep$ or insomnia

43. Thrush

44. Weight loss or weight reduction or weight fluctuation$ or loss adj weight

45. Weight gain or weight increase

46. Xerostomia or dry mouth or oral dryness

47. Symptom$ or pathological conditions, signs and symptoms

48. Terms 15-47 were combined using OR

49. Results from the search conducted in numerals 14 and 48 were combined using AND

50. Prevalence

51. Incidence

52. Rate*

53. Terms

50-52 were combined using OR

54. Results from the search conducted in numerals 49 and 53 were combined using AND
