# Supplementary material for: Symptom prevalence of patients with fibrotic interstitial lung disease: a systematic literature review
Source: BMC Pulm Med. 2018 May 22;18:78. doi: 10.1186/s12890-018-0651-3 (PMC5964639; doi:10.1186/s12890-018-0651-3)
Supplement: Supplementary file 2 — Appendix B Data extraction form. (DOCX 13 kb) [file 12890_2018_651_MOESM2_ESM.docx]

Additional file 2 APPENDIX B Data extraction form

| Study | Year | Setting | Type of Participants | Diagnosis Criteria | Number of Participants | Aim | Design | Measurement methods | Symptom prevalence | Notes |
| --- | --- | --- | --- | --- | --- | --- | --- | --- | --- | --- |
|  |  |  |  |  |  |  |  |  |  |  |
|  |  |  |  |  |  |  |  |  |  |  |
|  |  |  |  |  |  |  |  |  |  |  |
|  |  |  |  |  |  |  |  |  |  |  |
