# Supplementary material for: Symptom prevalence of patients with fibrotic interstitial lung disease: a systematic literature review
Source: BMC Pulm Med. 2018 May 22;18:78. doi: 10.1186/s12890-018-0651-3 (PMC5964639; doi:10.1186/s12890-018-0651-3)
Supplement: Supplementary file 3 — Appendix C Potentially Relevant but Excluded Studies. (DOCX 12 kb) [file 12890_2018_651_MOESM3_ESM.docx]

Additional file 3 Appendix C Potentially relevant but excluded studies

Bajwah S, Higginson IJ, Ross JR, Wells AU, Birring SS, Riley J, et al. The palliative care needs for fibrotic interstitial lung disease: a qualitative study of patients, informal caregivers and health professionals. Palliat Med. 2013;27(9):869-76. Epub 2013/07/26.

Schoenheit G, Becattelli I, Cohen AH. Living with idiopathic pulmonary fibrosis: an in-depth qualitative survey of European patients. Chron Respir Dis. 2011;8(4):225-31. Epub 2011/08/23.
